# Getting to Know a Place: Built Environment Walkability and Children’s Spatial Representation of Their Home-School (h–s) Route

**DOI:** 10.3390/ijerph14060607

**Published:** 2017-06-06

**Authors:** Mika R. Moran, Efrat Eizenberg, Pnina Plaut

**Affiliations:** Faculty of Architecture and Town Planning, Technion—Israel Institute of Technology, Haifa 3200003, Israel; eeizenberg@gmail.com (E.E.); pninatech@gmail.com (P.P.)

**Keywords:** walkability, built environment, spatial knowledge, children, school travel mode

## Abstract

The literature on environmental walkability to date has mainly focused on walking and related health outcomes. While previous studies suggest associations between walking and spatial knowledge, the associations between environmental walkability and spatial knowledge is yet to be explored. The current study addresses this lacuna in research by exploring children’s mental representations of their home-school (h–s) route, vis-à-vis objectively measured environmental attributes along the actual routes. Ninety-two children aged 10–12 years old (5th and 6th graders) drew sketch maps depicting their h–s route and drew the actual route on a neighborhood map, in addition to completing a brief survey. h–s routes went through Geographic Information Systems (GIS) analysis, yielding an en-route walkability index and its components. Children in traditional neighborhoods outperformed in the route’s orientation and structure, but not in the richness of the drawn maps. The orientation and structure of the drawn routes was related to objectively measured walkability, density, street connectivity and commercial land-uses along h–s routes. These associations remained significant among children who walked to school, but not among those who were driven to school. These findings highlight the importance of urban form and school travel mode in acquiring navigation skills and getting to know one’s neighborhood.

## 1. Introduction

### 1.1. Spatial Knowledge

Spatial knowledge is crucial for humans’ orientation and wayfinding capacities that are associated not only with functioning well in the environment, but also with psychological comfort, sense of competence and security [1,2,3]. Based on seminal works [4,5,6,7], research on spatial knowledge has developed to form a well-established body of literature. Nevertheless, there are still some competing and not necessarily complementary notions that this article tries to confront in its search for a better understanding of the associations between spatial knowledge, physical environmental (design and layout), and travel behavior.

According to Heft ([8], p. 22): “Human beings are equipped with an abstract framework that, among other things, makes it possible to adopt a point of view that is not normally attainable for a terrestrial organism, namely, a view of the earth’s surface as seen from ‘above’, as if it were a cartographic map”. The mental representations of environments stemming from this abstract framework are embodied in “cognitive maps” [9]. The formulation of this mental representation requires the mental processing and manipulating of myriad pieces of information, especially when concerning a large-scale environment, such as a neighborhood or a city [10].

Our spatial knowledge is seen as comprised of two distinct skills—“declarative” (sometimes referred to as “recognition”) and “operational” (sometimes referred to as “practical”) spatial knowledge [11]. Operational spatial knowledge, also referred to as “wayfinding”, is the capacity to actually navigate within a “real world” environment. It is “the interactive, problem-solving process by which people use environmental information to locate themselves and navigate from place to place” [11] (p. 189). Declarative spatial knowledge is the ability to recognize environmental cues, such as landmarks, and to locate ourselves on a map, plan our routes, and create representations of the environment.

While many studies on spatial knowledge have focused on newly learned environments and operational spatial knowledge, the current study focuses on highly familiar environments and the development of declarative spatial knowledge among children. The following subsections begin by reviewing various factors that are known to be related to spatial knowledge, proceed into a brief description of sketch maps as a mean to assess spatial knowledge, and resume by presenting the current study.

### 1.2. Gender Differences in Spatial Knowledge

Accumulating empirical evidence supports associations between background variables, such as age and gender, and spatial knowledge. Spatial knowledge is a developing rather than fixed construct; as such, it increases with age during childhood through early adulthood [12,13] and decreases during older ages [14]. Overall, children develop wayfinding abilities equivalent to older adults’ abilities at about the age of twelve [12,13]. However, beyond developmental factors, spatial knowledge is also shaped by other factors that increase with age during childhood, such as the intensity and quality of experiencing the environment as manifested through activity and travel behavior (see Section 1.4).

In contrast to the common perspective that men outperform women in orientation skills, past research has shown mixed results. In a review of the literature, Coluccia and Iosue [15] show that 61% of the studies that were reviewed found better performance in men, while the remaining studies showed no gender difference. It has been suggested that men are typically better at using configurational data, while women are better at using landmark information [16,17]. For example, Galea and Kimura [17] found that men were more capable of learning new routes from a map, while women performed better in recalling landmarks. In another study [18] that included a route navigation task among college students, men performed better in wayfinding efficiency and directional accuracy, but no gender difference were observed in the choice of route back. In addition, some studies found that women estimated their own orientation capacity much lower than the male participants, regardless of their performances [4,19].

Studies conducted among children are consistent with those among the adult population, suggesting that boys outperform girls in wayfinding, while girls are better in remembering landmarks. For example, in an experiment that was conducted among fifty-one children aged 5 and 12 and included retracing a route from a newly shown map [20], boys were better in retracing a route from a newly shown map, but girls recalled more landmarks than boys. These patterns of findings were observed in other studies among children [21].

### 1.3. Spatial Knowledge and the Built Environment

The associations between spatial knowledge and the built environments has been well researched. Lynch’s [1] comprehensive framework of physical features explains the legibility of place and its impact on wayfinding through five elements of the city: (1) “path”—a channel by which transport takes place (e.g., streets, trails), (2) “landmark”—a well-known/noticeable place (e.g., statue, building), (3) “edge”—a linear element separating one place from another (e.g., walls, railroads), (4) “district”—an area with distinct common characteristics (e.g., residential neighborhood, college campus), and (5) “node”—a junction between other elements (e.g., street intersections, town square).

Based on Lynch’s framework, Gärling et al. [10] conceptualized three design elements that are essential to legibility and wayfinding: (a) “visual access” (the visibility of landmarks and related attributes), (b) “differentiation of environments”, and (c) “simplicity of layouts”. Cubukcu and Nasar [22] examined the acquisition of spatial knowledge in largescale virtual environments, and found that landmarks and road differentiation contribute to acquired spatial knowledge. However, at the same time they found that simple design with fewer navigation choices “will help people learn their way around” [22] (p. 397). From here it may be assumed that the ability to understand the environment is challenged by both overly complex and overly simple environmental layouts.

Further research has established that differentiated physical structures in the environment and particularly landmarks are central to the development of spatial operational knowledge [3,23]. In the case of children, landmarks that function as a part of an activity (e.g., statues that can be climbed on) play a more significant role in spatial representation than landmarks that are visually more salient [2].

The lack of research studies regarding how specific environmental attributes aid in the construction of spatial representation was noted three decades ago [24], but very little has been done since to fill the void. Nevertheless, Golbeck [24] provided a useful taxonomy of physical environmental features, distinguishing between structural and organizational features. According to Golbeck, the structural features, such as landmarks, roads, and paths, are objective and easy to measure and compare. The organizational features, such as clusters, orientation, and saliency of environmental features are central to our knowledge of the environment but much harder to objectively measure.

### 1.4. Spatial Knowledge, Travel Behavior and the Built Environment

Activity, known as travel behavior, is acknowledged as a significant factor in the acquisition of spatial knowledge [25]. Various social and environmental factors (conceptualized as types of individual-environment interactions) influence activity and, in turn, shape our spatial knowledge and skills [26]. Correspondingly, these social and environmental factors are the targets of most previous and contemporary studies on spatial knowledge and its mental representation.

“Freedom of movement” or “autonomy” is particularly important to determine children’s experiences of their environment and the type of activities they can employ [25,26]. Thus, by examining children’s mobility in their environments, research distinguishes between different modes of transportation (e.g., driven by car, walk with an adult, or walk alone), concluding that walking-on-their-own yields better declarative spatial knowledge [27,28]. Pedestrianism is the mode of transportation with the “most direct sensorimotor experiences” ([25], p. 204). In addition to the contribution of the direct experience of walking within a given environment, Cohen and Cohen [25] claim that in regard to larger-scale areas (e.g., city, district), our spatial knowledge is also highly enhanced by driving across extended areas. In the case of children, distance also has an indirect effect on spatial knowledge because distance determines the travel mode to school, as shorter distances may be associated with more walking, and thus yield better spatial knowledge of the route. Indeed, Ahmadi and Taniguchi [27] found that distance had an indirect effect on children’s spatial representation accuracy, as shorter travel distances to school were associated with more walking, which, in turn, was found to be associated with more accurate spatial representations.

Travel behavior and particularly walking have been shown to be associated with the built environment. Results of research to date indicate that walking is positively related to environmental walkability defined as overall support for pedestrian and cyclist travel and marked by: residential density, streets connectivity and land-use mix [29,30]. Walking to school was also found to be associated with environmental walkability [31,32] as well as to other environmental features, such as residential density, street connectivity and route distances [6,33,34].

Given the vast literature linking walking with environmental walkability [30,31,35,36,37] on the one hand, and with spatial knowledge [27,28], on the other hand, it is likely to expect that environmental walkability will be associated with spatial knowledge. And indeed, some theoretical work [38,39] have linked between the concepts of environmental walkability and legibility, which, in this context, serves as an isomorph of spatial knowledge. However, to the best of our knowledge, the associations between environmental walkability and spatial knowledge have not yet been examined empirically. The current study was intended to address this gap, as described below.

### 1.5. Assessing Spatial Knowledge through Sketch Maps

Declarative spatial knowledge is usually examined through different forms of representations, such as: recognition tasks, drawing routes on blank maps and sketch maps. Sketch maps, also known as mental or cognitive maps, represent one’s topographical memory or mental representation of the different aspects of the environment that were processed. According to Campbell et al. [40], topographical memory includes “the ability to recognize/identify landmarks, the ability to remember their locations, the ability to judge current heading orientation and the ability to navigate or describe a route” [40] (p. 1). The use of sketch maps was criticized for remaining a positivist framework aiming at quantifying and generalizing spatial knowledge. The critique suggests that research using sketch maps should turn to critical knowledge production and positionality understanding [41,42]. However, of the different methods used in spatial knowledge research, sketch maps were found to be highly reliable [43,44]. This is particularly relevant in the case of children, although they may differ in their drawing capacities [45,46].

In previous studies, sketch maps outcomes were used to assess children’s spatial knowledge in relation to travel mode and other background variables. Rissotto and Tonucci [28] used sketch mapping with 8–11 years old children (n = 46), who lived in Rome (Italy), to examine the influence of school travel mode on the orientation and structure performance. Ahmadi and Taniguchi [27] used a sketch map and route drawing on a blank map to distinguish between influential factor on spatial knowledge with an emphasis on mobility of children aged 9–13 (n = 75) in Teheran (Iran).

Holt et al. [47] integrated mental maps with GIS-based walkability data in a study conducted in the city of Edmonton (Canada). This study examined maps drawn by 168 children aged 6–12, who live in high- and low-walkable neighborhoods, as defined by residential density, street connectivity, and land use mix. The children’s drawn maps went through thematic analysis, in which images were coded into themes. According to the findings, children from the high-walkable neighborhood depicted more play space for physical activity than do those from low-walkable neighborhoods [47]. While this study is amongst the firsts to combine cognitive mapping with objective environmental walkability data, its’ findings merely focus on the content of drawn maps, rather than on their spatial orientation and structure, which may reflect children’s wayfinding capabilities. The current study addresses this gap in literature by analyzing both the content and orientation and structure of children’s sketch map, while examining their associations with objective walkability measures.

### 1.6. Current Study

The review of the literature alludes to several gaps within existing understanding of spatial knowledge that this study sought to overcome by examining the associations between children’s spatial knowledge, environmental walkability and travel behavior. By doing this, the current study addresses a lacuna in the literature that was recently recognized by Vandenberg et al. [11], who called for more studies exploring the associations between the built environment, walking and wayfinding. A series of research objectives and corresponding hypotheses were developed, as follows:

1. To explore children’s spatial knowledge and representation of their h–s route as obtained through their drawn sketch maps (henceforth: maps’ summary scores). Following previous studies [28,29] we distinguished between the map’s orientation and structure, and its richness, which reflect the number of details drawn in the map.
2. To examine the associations between the maps’ summary scores and built environment attributes at both neighborhood and route scales. Based on the vast literature on walking and environmental walkability on the one hand, and spatial knowledge, on the other hand; we hypothesized that environmental walkability and its components will be positively associated with spatial knowledge (as represented by the maps’ scores).
3. To examine the association between maps’ summary scores and school travel mode. Based on existing literature, we hypothesized that children who walk to school would have better spatial knowledge and representation of the h–s route than those who don’t.
4. To examine the association between maps outcomes and gender. Based on the mixed evidence in prior research on gender and spatial knowledge, no hypothesis was made.

To assess the independent contributions of each of the aforementioned variables—built environment, school travel mode and gender on children’s spatial knowledge (as represented by their maps’ summary scores). This objective had a more exploratory nature and thus was not designed to test a specific predetermined hypothesis.

## 2. Materials and Methods

### 2.1. Study Area

**Figure 1:**
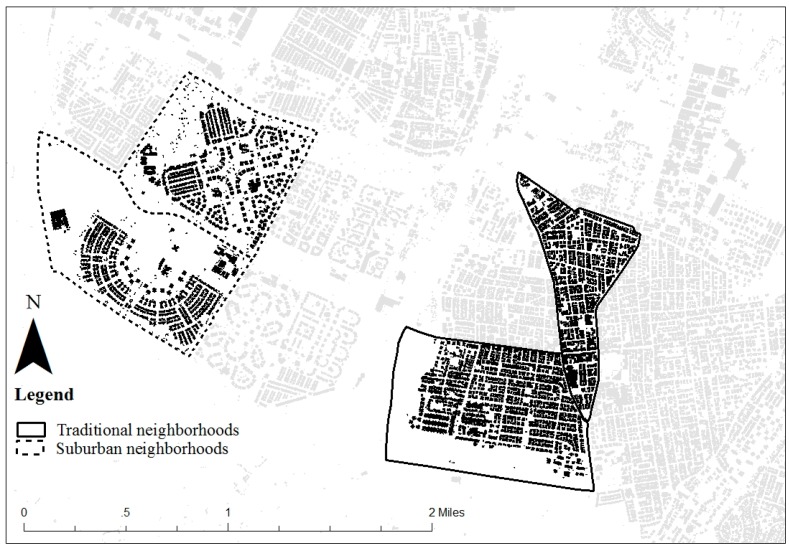
Figure ground of the study area.

**Figure 2:**
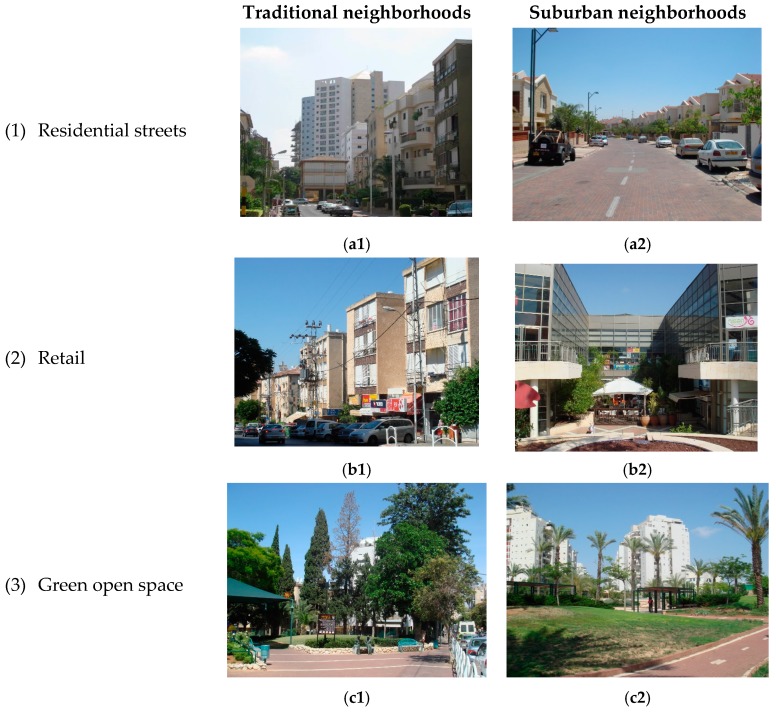
Urban design in traditional and suburban neighborhoods (**a1**) residential street in a traditional neighborhood, (**a2**) residential street in a suburban neighborhood, (**b1**) retail street in a traditional neighborhood, (**b2**) a mall in a suburban neighborhood, (**c1**) green open space in a traditional neighborhood, (**c2**) green open space in a suburban neighborhood.

The study was conducted in the city of Rishon LeZion, the fourth largest city in Israel (228,200 inhabitants), located along the central Israeli coastline plain, 12 km south of Tel Aviv. Four neighborhoods were selected to include two types: “traditional neighborhoods” (N = 2), characterized by high density, grid street network, land-use mix and high access to commercial destinations, and “suburban neighborhoods” (N = 2), characterized by low density, land-use segregation, cul-de-sac streets and abundant green open space. All 4 neighborhoods are located within the city of Rishon LeZion. A figure ground map of the study area (Figure 1) shows the differences in urban texture between the traditional and suburban neighborhoods. Additional street-level differences between the two neighborhood types are presented in Figure 2.

As shown in Figure 2, the residential streets in the traditional neighborhood (Figure 2a1) are characterized by human-scale design, consisting of a narrow road, short road-to-building distance and street-facing balconies, while in the suburban neighborhood the residential streets (Figure 2a2) are characterized by automobile-oriented design, including a wide road and a longer road-to-building distance, which disconnect the houses from the street. Similarly, the retail street in the traditional neighborhood (Figure 2b1) is pedestrian-oriented allowing access to street-facing stores with shaded stores fronts, while in the suburban neighborhood the shops are located within a mall (Figure 2b2). The differences between the two neighborhood types are also manifested in their green open spaces. While the green open space in the traditional neighborhood (Figure 2c1) is relatively small and surrounded by other daily destinations (e.g., bus-stop, stores, school), the green open space in the suburban neighborhood (Figure 2c2) is spread on a large area surrounded by residential land-uses.

The two areas (traditional and suburban) were chosen because they have similar socio-economic indicators, including the percent of participants in labor force (97.3–98.4% in traditional neighborhoods, and 97.4–98.8% in suburban neighborhoods), and the percent of recipients of an undergraduate academic degree (27.8–31.4% in the traditional neighborhoods, and 23.4–30.7% in the suburban neighborhoods). Additionally, these neighborhoods do not have public housing on their premises. The city of Rishon LeZion was chosen for the current study due to its aforementioned homogenous population on the one hand, and heterogenous built environments on the other hand. These conditions enable investigation of the potential impact of urban design on spatial knowledge, while a priori controlling for socioeconomic factors.

From a broader perspective, data on activity patterns and safety reveal some similarities and differences between the two neighborhood types. According to a previous research in the study area among 573 children (aged 10–12) [48], Walking (to school and to neighborhood destinations) was more common in traditional neighborhoods, while biking (for travel and leisure) was more common in suburban neighborhoods. Overall, 69.5% of the city’s children reported walking to school most of the week at least four out of six school days, 54.9% reported walking to neighborhood destinations at least three times a week, 51.6% reported biking for leisure at least once a week, and 35.2% reported biking to neighborhood destinations at least once a week.

Regarding the traffic situation in the study area, private car ownership in the city of Rishon LeZion is higher than the national average (447 versus 365.5 per 1000 residents on average) [49], and the public transit use (as measured by mouthy household expenditure) is the lowest among Israeli large cities (with a population over 100,000) [50]. Previous findings from the current study area [51], revealed no differences between traditional and suburban neighborhoods in terms of children’s road safety perceptions. Of the 573 children (10–12) participating in the study, 56% reported having heavy traffic passing through their neighborhood, and 39% described traffic is a barrier for neighborhood walking [51].

The sense of security in Rishon Lezion was found to be the highest among large Israeli cities (cities with a population over 100,000), as 79% of the city’s residents reported feeling safe walking alone in their neighborhood at night [52]. Sense of security was also examined among children in our study area in the aforementioned previous study [48], showing that most of the children aged 10–12 (76%) feel safe walking alone during daytime and 28% feel safe walking alone at night. No difference in children’s sense of security were observed between the two neighborhood types [51].

### 2.2. Participants and Procedure

Participants were recruited from four primary schools in the study area (one school within each neighborhood). Overall, 92 children aged 10–12 years old (5th and 6th graders) participated in this study, 52 of whom live in traditional neighborhoods and the remaining 40 live in suburban neighborhoods. We chose to focus on this age group because 10–12 years old children have enough freedom of movement to walk or bicycle to school on their own, unlike younger children who are more confined to their home surroundings and do not walk around alone (by law, children under the age of 9 are not allowed to cross street alone). Furthermore, by around these ages, children develop wayfinding abilities equivalent of adults’ abilities [12,13].

A similar number of boys and girls participated in the study (43 and 49, respectively). Of the 52 children who live in traditional neighborhoods, 22 were boys and 30 were girls, while of the 40 children who live in suburban neighborhoods, 21 were boys and 19 were girls (χ^2^(1, n = 92) = 0.95, *p* = 0.22). Nearly two thirds of the sample (n = 59) consisted of 5th graders, and the remaining 33 children were 6th graders. Fifth and 6th graders were equally distributed in traditional neighborhoods (27 and 25, respectively), while most of the participants from suburban neighborhoods (32 out of 40) were 5th graders (χ^2^(1, n = 92) = 5.67, *p* = 0.01). Given that the length of residence may be related to one’s spatial knowledge, children who were new in their neighborhood (moved in within a year before the survey) were not included in the study.

Ethics approval for this study was received from the university Ethics Committee and from the Israeli ministry of Education. Prior to data collection, school principals and teachers were provided with information regarding the study, and consent forms and information regarding the study were delivered to the children’s parents through the school. Children participated in this study only after providing their parents’ consent and their own assent. It was clearly stated that the data would be analyzed anonymously and that the participant could withdraw from the study at any stage. Data were collected during school days during May–June 2011.

Within each school, 5th and 6th graders were recruited to participate in a sketch map activity and to complete a brief survey. Sketch map drawing was used to describe children’s representation and knowledge of their h–s route. Children were provided with a white, blank paper sheet size A4 (210 × 297 mm), and were briefly instructed as follows: “Please draw a map of the route along which you walk from home to school and what you see along the way”. No further instructions were given, except for clarifications as needed. For example, in a few cases, when needed, it was clarified that the drawing should be made from an aerial perspective rather than a terrestrial perspective. These minimalist instructions were followed by minimal communication between the researcher and the participants that served to ensure that the sketch map drawing task will be done as authentic as possible and to avoid potential biases that may otherwise be introduced. After completing the sketch maps, children were provided with a street map of their neighborhood, upon which they were asked to mark the school location, their home address, and their h–s route. The children also completed a brief survey concerning their school travel mode and socio-demographic characteristics. This activity lasted approximately 30 min, occurred during school hours in small groups of up to 7 children and was facilitated by the first author. Although the mapping activity was conducted in small groups, each participant completed the procedure individually. To avoid interactions and mutual influences among participants, it was clearly stated that this was an individual activity that each participant needs to complete on his/her own.

### 2.3. Constructs and Measures

This study included two dependent and four independent variables as listed in Table 1. It is noteworthy that additional demographic variables (age, number of siblings and birth-order in the family) were also included in the survey, but were not found related to the study’s variables, and thus are not reported here.

Data were analyzed using three different approaches as follows: First, sketch maps analysis was used to evaluate children’s spatial knowledge and representation. Then, GIS analysis was employed to assess the built environment along the actual h–s routes. Finally, a correlation analysis was conducted to identify associations between the children’s spatial knowledge and representation with the independent variables, including objective GIS measures of the built environment, school travel mode and gender. It should be noted that additional self-reported data on independent mobility and perceived environment obtained in a previous study [48,51] was analyzed to identify associations with spatial knowledge as measured in this study. However, since no associations were observed, these findings are not reported.

*Sketch maps scoring schemes*. Children’s sketch maps of their h–s route were analyzed in order to assess their spatial knowledge and representation. For this analysis, the knowledge of the route refers to the extent to which one knows how to navigate through his/her surroundings, and the representation of the route refers to the extent to which one is acquainted with his/her neighborhood surroundings. This analysis was based on the orientation-structure analysis method developed by Rissoto and Tonnuci [28], which includes three aspects: The orientation of the route, structure of the route and level of details. While Rissoto and Tonnuci [28] examined these three aspects separately, we combined the analysis of orientation and structure to reflect children’s spatial knowledge of the route in this study. We then conducted a separate analysis to assess the map’s richness by quantifying the level of detail in the map. Correspondingly, two scoring schemes were developed, one of which focuses on the accuracy of the route (i.e., orientation and structure), and the other focuses on the richness of the map (i.e., diversity and level of detail). The children’s maps were scored by a trained rater. To ensure the reliability of the scoring schemes, a sub-sample of maps (n = 30) was also scored by the first author, and yielded acceptably high score inter-rater reliability, as detailed below.

*Orientation and Structure summary score*. In order to assess the participants’ knowledge of the route, the sketch maps were compared with the actual route maps (obtained by the children as part of the activity). Based on this comparison, orientation and structure scores were calculated as follows: (1) Orientation score: The orientation of the route was assessed by the home position with regard to the school position in the sketch maps compared to that in the actual route map. An orientation score was developed ranging from 0 to 2, while 0 = inaccurate orientation (in terms of both top/down and right/left literalities), 1 = partially oriented map (one laterality is accurate (either top/down or right/left), 2 = accurate orientation (in terms of both top/down and right/lest literalities). (2) Structure score: The structure of the route was assessed by subtracted the number of segments in the sketch maps from that in the actual route. For the sake of analysis, the gap in segments in the sketch map versus reality was converted into absolute values, thus yielding a structure score ranging from 0 to 8, when 0 = accurate structure (the number of segments in the sketch map is the equal to that in the actual map), 1 = there is a difference of one segment between the sketch map and the actual map, … and 8 = inaccurate structure (there is a difference of 8 segments between the sketch map and the accurate map). Both orientation and structure scores yielded high inter-rater reliability (r = 0.96, n = 30, *p* < 0.0001; and r = 0.91, n = 30, *p* = 0.0012, respectively).

The orientation and structure scores were combined in order to evaluate the overall accuracy of the map. For this analysis, the values of the structure score were flipped, and the two scores (the orientation and flipped structure score) were then summarized, yielding a variable ranging from 1 to 10, when: 1 = inaccurate map, and 10 = accurate map. A correlation analysis between the map’s accuracy score components yielded results as expected, suggesting that the better the map is oriented the smaller the gap in the routes segments between the map and the actual route (r = −0.21, *p* = 0.04, n = 92). Although this relatively low correlation coefficient might indicate limited construct validity, we decided to use the orientation and structure summary score given evidence from previous studies using similar measures [27,28].

*Richness summary score*. The diversity and level of detail of the sketch map were evaluated, assuming that the number and variety of objects drawn reflect the participant’s familiarity with their environment. First, all of the elements in the maps were coded, and then they were categorized into different themes to reflect the diversity of elements drawn. Six themes and corresponding elements were identified as follows: pedestrian infrastructure (sidewalk, pedestrian path), car infrastructure (junction, parking lot, zebra-crossing), public transit (bus stop), recreational space (park, playground), greenery (grass, tress), commercial destinations (retail shops, malls). This analysis resulted in two scores: (1) elements score (reflecting the maps’ level of detail): this score consisted of the number of different elements drawn in the map—ranging from 0 to 13, when 0 = no elements in the map (except the home, school and the route), and 13 = 13 different elements were drawn in the maps (in addition to the home, school and the route); (2) themes score (reflecting the maps’ diversity): this score consisted of the number of different themes in the map—ranging from 0 to 7, when 0 = no themes in the map (except the home, school and the route), and 7 = elements of seven different themes were included in the map. Both elements and themes scores yielded high inter-rater reliability (r = 0.89, n = 30, *p* = 0.006; and r = 0.93, n = 30, *p* = 0.002, respectively).

In order to evaluate the overall richness of the map, the themes and elements score were summarized, yielding a variable ranging from 0 to 16, where: 0 = sparse map (including no theme/element except the home, school and the route) and 16 = rich map (including an overall of 16 different themes and elements). Correlation analysis between the maps richness score components yielded results as expected, indicating high construct validity, as the more elements included in the map, the higher number of different themes were identified in the map (r = 0.79, *p* = 0.0001, n = 92).

*GIS-based environmental variables along h–s routes*. Objective measures of the built environment along the actual h–s routes were obtained through GIS analysis (ArcGIS 9.3, ESRI, Redlands, CA, USA). First, A GIS shape file of the h–s routes was created according to the schools’ and participants’ addresses, and the routes maps obtained by children. Then, the following environmental variables were measured along the route within a 25-m buffer area. GIS data (from 2009) was provided by the Municipality of Rishon LeZion.

*Walkability index*. We used the walkability index that was developed by Frank et al. [29], and incorporates land use mix (as measured by the entropy index) with urban form attributes, including: residential density, intersection density, and retail floor area ration. The walkability index was chosen for this study due to its well-established association with travel walking [53] and particularly with children’s walking to school [31,32]. Based on these associations, along with the associations between travel walking and spatial knowledge [27,28], it was hypothesized that high values of environmental walkability would yield a more legible environment and thus would be related to better spatial knowledge (as manifested in higher accuracy scores).

*Urban form variables*. Two urban form variables were calculated as follows: (1) residential density—number of households per sq km and (2) street connectivity—number of intersections per sq km. These variables were calculated within a 25-m buffer along the route. Given that these two variables are components of the walkability index [29], it was hypothesized that higher values of residential and intersection densities would yield a more legible environment and thus would be related to better spatial knowledge (as manifested in higher accuracy scores).

*Land use variables*. Three land use variables were calculated, consisting of the proportion of land area along the route (within a 25-m buffer along the route) dedicated to the following: (1) retail, including shops, grocery stores, malls etc., (2) public institutes, including community centers, recreation facilities etc., and (3) green open space—including parks, playgrounds etc. These variables were calculated as the percent of land within the buffer area dedicated to each use. It was hypothesized that higher access to these non-residential destinations would yield a more legible environment and thus would be related to better spatial knowledge (as manifested in higher accuracy scores). This hypothesis is based on existing theoretical frameworks [1,10], while assuming that non-residential destinations can be perceived as landmarks and thus add to the differentiation of the environment.

The rational for using both the walkability composite measure and it’s components (intersection density, residential density and land uses en-route) lies in the environmental characteristics of the h–s route in the study area (Table 2). Although the walkability index of routes in traditional neighborhoods was higher compared to that of those in suburban neighborhoods, when looking at the walkability index components individually, inconsistent patterns were revealed. While the values of en-route residential density and retail land uses were higher in traditional neighborhoods, the value of en-route green open spaces was higher in suburban neighborhoods. From here we deliberately chose to include in our analysis both the walkability index and its components, as according to our data, the index alone may not reflect all of its components.

Table 2 shows descriptive statistics of the environmental measures along the h–s routes and provides several differences between traditional and suburban neighborhoods. As shown in Table 2, compared to those in suburban neighborhoods, h–s routes in traditional neighborhoods are characterized by higher overall environmental walkability (1.14 vs. −0.83, *p* < 0.0001), higher levels of residential density (21.61 vs. 7.42 households per sq km, *p* < 0.0001), similar intersection density (difference not significant), and a higher proportion of retail land uses (8% vs. 0% of the land along the route, *p* < 0.0001). Green open space, on the other hand, was more common along h–s routes in suburban neighborhoods (19% vs. 2% of the land along the route, *p* < 0.0001). No differences were observed between the two neighborhood types in terms of the proportion of the proportion of land used for public institutes en-route. Finally, h–s routes in suburban neighborhoods were longer than those in traditional neighborhoods (M = 570 vs. M = 410 m, respectively, *p* = 0.001). This difference in route length might imply that children from suburban neighborhood choose longer routes to their school. However, the longer h–s routes in suburban neighborhoods might be attributed to the design in those neighborhoods, which consists of larger lots and lower street connectivity, thereby creating longer distances.

### 2.4. Analysis Plan

Statistical analyses were performed using SPSS version 21.0 (IBM Corporation, Armonk, NY, USA). First, conventional descriptive statistics were used to describe the sketch maps’ summary scores (aim 1), and chi-square analysis was conducted to compare between neighborhood types (aim 2), school travel mode (aim 3), and gender (aim 4). Pearson correlation analysis was used to examine associations between the sketch maps’ summary scores and environmental variables en-route to school (aim 2). Finally, multivariate linear regression analysis was conducted to determine the best joint predictors of sketch maps’ summary scores (aim 5). For correlation and regression analyses, the outcome variables were recalculated by using Zscores of the variables components. Conventional level of *p* ≤ 0.05 was taken to represent statistical significance.

## 3. Results

**Figure 3:**
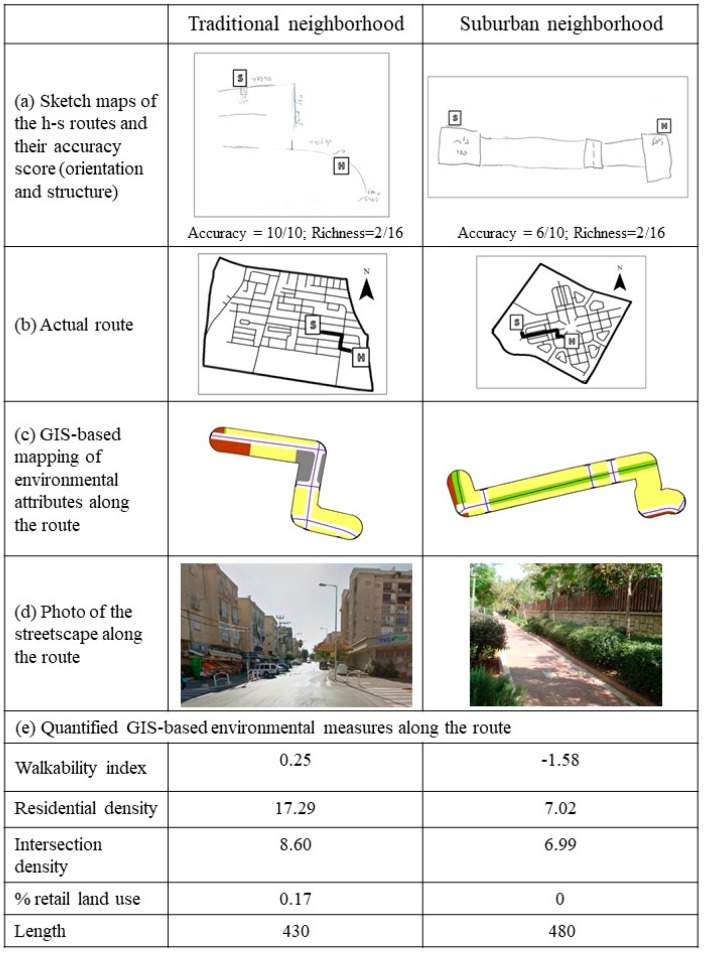
(**a**) Sketch maps of home-school routes and their accuracy score (orientation and structure), (**b**) Maps of the actural routes, (**c**) GiS based mapping of streets and land uses along the routes, (**d**) Street view photos of the routes, (**e**) Quantified GIS-based environmental measures along the route.

Figure 3 illustrates the analysis procedure and its intermediate outcomes, by presenting two typical sketch route maps and their scores (Figure 3a), as well as the actual routes characteristics, including: maps of the actual routes (Figure 3b), GIS data as measured within a 25 m buffer along the routes (Figure 3c), street view photos of the routes (Figure 3d), and quantified GIS-based environmental variables along the route (Figure 3e).

Table 3 presents the sketch map scores in the total sample and by neighborhood type, school travel mode and gender. Overall, children drew relatively accurate maps with an average orientation and structure summary score of 8.23 out of 10. Regarding the richness of the maps, children’s maps were relatively minimalistic in terms of their diversity and level of detail, with an average of three elements and two themes per drawing. However, this might be attributed to the instructions given to the children prior to the mapping activity: children were asked to draw the route, rather than to draw the routes’ surroundings.

**Figure 4:**
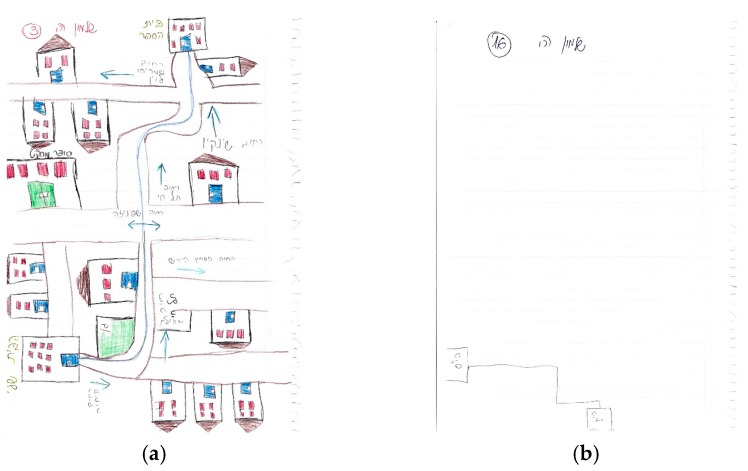
(**a**) a drawn sketch map with a high richness score (10/16), (**b**) a drawn sketch map with a low richness score (0/10).

Figure 4 further provides examples of high and low richness scores, both of which were drawn in the same traditional neighborhood. It is noteworthy that most of the maps were sparse and had low scores (3 or lower), and the rich score presented in Figure 4 is not representative.

### 3.1. Sketch Maps’ Summary Scores in Traditional and Suburban Neighborhoods

As presented in Table 3, the accuracy of h–s route was better among children from traditional neighborhoods. The sketch maps’ accuracy scores were significantly higher in traditional compared to suburban neighborhoods (8.81 vs. 7.48). This difference is attributed mainly to the structure component, as the proportion of children who drew the same number of segments as in the actual route was much higher in traditional versus suburban neighborhoods (62% vs. 15%, χ^2^(1, n = 92) = 20.20, *p* < 0.0001). Similarly, a higher proportion of children drew well-oriented maps in traditional neighborhoods versus suburban neighborhoods, yet these differences were not found significant (69% vs. 55%, χ^2^(1, n = 92) = 1.97, *p* = 0.12). No differences were observed between the two neighborhood types in terms of the richness scores of the maps (reflecting the number of elements and themes drawn in the map). However, the specific elements included in sketch maps differed between the two neighborhood types, in a way that corresponds with the actual environment in the two neighborhood types. Specifically, malls were more commonly drawn in maps from suburban neighborhoods (11% vs. 1%), while retail shops were more common in maps drawn by children from traditional neighborhoods (20% vs. 3%). This difference well reflects the study area as retail stores are more common in traditional neighborhoods, while malls are more common in suburban neighborhoods. The differences between the two neighborhood types are well-manifested in Figure 3a, which presents a map from a traditional neighborhood that obtained a high accuracy score (10 out of 10) versus a map from a suburban neighborhood that obtained a low accuracy score (six out of 10). Both maps obtained low richness scores (two out of 16 for both maps). Specifically, except for the home and school, the highly accurate map included some street names, and the inaccurate map included a zebra-crossing en-route.

### 3.2. Associations between Sketch Maps’ Summary Scores and Environmental Attributes along h–s Routes

Correlation analyses were conducted to identify GIS-based environmental variables along the routes to school that are related to sketch maps accuracy and richness scores (Table 4). Overall, the environmental attributes en-route were found to be significantly related to the accuracy score but not to the richness score. The sketch map accuracy score was positively correlated with the walkability index (r = 0.40, *p* < 0.0001), residential density (r = 0.35, *p* = 0.014), intersection density (r = 0.30, *p* < 0.0001), and percent of retail area en-route (r = 0.22, *p* = 0.03). A negative correlation was observed between the accuracy score and the percent of green open space area en-route (r = −0.21, *p* = 0.03). However, this negative correlation may be attributed to the overall low accuracy scores in suburban neighborhoods, where parks are more accessible. Indeed, this correlation disappeared when examining traditional and suburban neighborhoods separately.

Figure 3 illustrates the aforementioned correlations between sketch map accuracy scores and GIS-based environmental features of h–s routes. Figure 3 row a presents a comparison of highly accurate versus inaccurate map (scoring 10 versus six out of 10), obtained by a child from a traditional and a child from a suburban neighborhood, respectively. According to the environmental analysis along those routes (Figure 3e), the route in the traditional neighborhood was characterized by higher values of environmental walkability, residential and intersection density and a higher proportion of retail and public institute land uses.

In addition to environmental attributes along the route, the route distance was significantly negatively correlated with the accuracy score (r = −0.43, *p* < 0.0001, n = 92), suggesting that children who walked shorter distances to school tended to draw their h–s route more accurately. This negative correlation was observed both in traditional (r = −0.40, *p* = 0.004, n = 52) and suburban neighborhoods separately (r = −0.45, *p* = 0.003, n = 40). No correlations between the route distance to the maps’ richness were observed.

### 3.3. Sketch Maps’ Summary Score by School Travel Mode

The accuracy scores obtained from maps of children who walk to school most of the week (at least four out of six school-days) were significantly higher than those of children who didn’t (M = 8.69 vs. M = 7.71, t(90) = −3.66, *p* < 0.0001). The richness scores of the sketch maps did not differ according to the children’s school travel mode.

Walking to school most of the week was more common in traditional compared to suburban neighborhoods (69% vs. 31%, χ^2^ = 12.80, *p* < 0.0001). However, after stratifying the sample according to neighborhood type, the association between maps accuracy and school travel mode was prominent in traditional but not in suburban neighborhoods. In traditional neighborhoods, 68% of the children who walk to school most of the week drew highly accurate maps (with accuracy scores of 9–10 out of 10), compared to 38% of the children who didn’t walk to school most of the week. However, in suburban neighborhoods, the percent of children who drew highly accurate maps was similar among those who walked and those who did not walk to school most of the week (45% and 40%, respectively). Further analysis aimed to explore environmental correlates of sketch maps scores among children who walk to school compared to those who do not. According to the results (not reported), the sketch maps accuracy scores remained significantly correlated with GIS-based environmental measures of the route among children who walk to school most of the week (at least four out of six schooldays) (n = 62), but not among those who were driven to school most of the week (n = 30). These findings may suggest that the impact of environmental attributes on spatial knowledge and representation is enhanced among those who walk to school. However, given the limited sample used for this study, these interactions could not be tested.

### 3.4. Sketch Maps’ Summary Score by Gender

In addition to the physical environment and school travel mode, sketch map accuracy and richness scores differed by gender (Table 3). While the sketch maps drawn by boys were better oriented and structured, those drawn by girls were richer in detail. These differences are manifested in the orientation and structure summary score, which was significantly higher among boys (M = 8.70 vs. M = 7.82, t(90) = −2.22, *p* = 0.03), and the richness score, which was borderline significantly higher among girls (M = 6.18 vs. M = 4.88, t(90) = 1.94, *p* = 0.06).

### 3.5. Multivariate Analysis to Predict Sketch Maps’ Summary Scores

Multivariate analysis was conducted in order to determine the environmental variables most strongly related to the orientation and structure summary score (study aim 5). Given the significant associations observed between neighborhood type and the environmental attributes along the h–s routes (Table 2), Neighborhood type (as a dichotomist variable) was the only environmental variable included in the multivariate linear regression model (Table 5). It is noteworthy that additional regression models were conducted by replacing “neighborhood type” with each of the environmental variables independently. However, in these models, none of the environmental variables were found significantly related to the outcome variable, and thus the findings are not reported.

According to the findings, the associations between neighborhood type and the orientation and structure summary score were no longer significant after adjustment for gender, school travel mode, and route distance. The model explained 30% of the variance in the orientation and structure summary, with the route’s length being the strongest predictor (β = −0.33, *p* < 0.01), followed by school travel mode (β = 0.23, *p* < 0.05) and gender (β = 0.20, *p* = 0.05).

## 4. Discussion

This current study explored objective environmental correlates of children’s spatial knowledge and representation of their h–s route, while distinguishing between traditional and suburban neighborhoods. On a theoretical level, the link between environmental legibility and spatial cognition is well established [1,2], and recent attempts have established connections between environmental legibility and walkability [38,39]. However, to the best of our knowledge, the direct relationship between environmental walkability and spatial knowledge and representations had not yet been explored. Similarly, studies on children’s spatial knowledge studies focused mainly on walking [27,28], while ignoring the potential impact of the built environment. This study adds to existing literature by exploring how spatial knowledge (as represented by the map scores) may be shaped by both the built environment and walking. By doing this, the study addresses a lacuna in the literature that was recently recognized by Vandenberg et al. [11], who called for more studies exploring the associations between the built environment, walking and wayfinding.

This study’s contribution to the literature on walkability is particularly significant as most research on environmental walkability thus far has focused on its impact on walking and travel behavior [30,31,32,35,36,37], with a few studies dealing with other influences on the community [54] and on health-related quality of life [55]. However, to the best of our knowledge, this study is the first to explore the potential impact of environmental walkability indicators on spatial knowledge. To explore this relationship, we objectively measured environmental attributes along the actual route and examined them vis-à-vis route sketch map data. Such an inquiry and analysis yielded genuine insights into the relationships between urban spatial form and spatial knowledge and representations.

A major finding of our study concerns the differences between the two neighborhood types in terms of children’s spatial knowledge of their h–s route. According to our findings, the sketch maps that were drawn by children from traditional neighborhoods were more accurate than those drawn by children from suburban neighborhoods. This finding stands in contrast to the conclusion of Cubucku and Nasar [22] when comparing acquired spatial knowledge in simple and complex virtual environments. In their findings, simple environments (with fewer navigation choices) were better acquired by adult participants, whereas in our study intersection density was significantly positively correlated with accuracy scores. While learning a new environment, simplicity is an advantage (and probably more so in the case of a virtual environment). However, our study suggests that in highly familiar environment within similar levels of exposure (children who walked to school on a daily basis), the higher complexity in traditional neighborhoods is related to better declarative spatial knowledge, in terms of accuracy and orientation.

It is important to bluntly state that the children of the two neighborhood types represented the same level of richness, diversity and details of their environment in their maps. This measure provides a balanced baseline of children’s capacity to draw a sketch map and represent their environment on the same level for the two neighborhoods types. However, it also helps framing the discussion of differentiated environmental attributes around spatial cognitive skills (i.e., accuracy) rather than enriching and stimulating creativity.

The results confirmed previous research on the positive relationship between walking to school and the accuracy of routes spatial representations [27,28,56]. Compared with those who were driven to school, children who walk to school in the two neighborhood types produced more accurate representations of their route, as indicated by their sketch maps. However, our GIS analysis suggested that children in the two types of neighborhoods were exposed to different spatial forms and arrangements in the routes. These environmental differences in the walkability index, residential density, intersection density and retail versus open/green land uses played a role in the capacity of the children to produce accurate representations of their environment.

In line with the inconclusive literature on the role of gender and spatial knowledge [15,21], we have also observed that gender differences are related to the specific aspects of spatial cognition being examined. Boys scored better in the accuracy of the representations, and girls scored better in richness of representations. These findings support previous studies showing that boys outperform in wayfinding skills, while girls may be better in remembering landmarks [20,21]. It is possible to suggest that boys and girls perceive the environment differently and receive different environmental cues that influence both their representation of the environment and most probably their ability to navigate within it.

This study has some limitations. First, although the research question is of a causal nature—exploring how environmental, behavioral and demographic attributes contribute to spatial cognition—this study is cross-sectional and thus does not allow to identify causal relationships. The sample used here was limited, and thus may limit the results generalizability. However, the choice of one city and a narrow age range of the study population aimed to minimize intervening factors. Another possible bias may be attributed to the fact that most of the participants in suburban neighborhoods were 5th graders; however, this was less likely to affect the results as no differences were observed between 5th and 6th graders in terms of their maps’ accuracy nor richness. This study included only two non-environmental independent variables (namely school travel mode and gender), and does not control for the effect of other sociodemographic (e.g., ethnic background, parents’ education, socio-economic status) and psychosocial (e.g., parents’ relationships with neighbors, and social insecurity) variables that are known to be related to spatial cognition. Similarly, this study did not distinguish between children who walked to school on their own versus those who were accompanied by others, a factor that is also known to be related to spatial cognition. Another limitation is related to the time-space context, as the sketch maps used in this study in this study are representations of an environment produced by an individual at a given time, and thus may vary from one time to another. In addition, the maps drawn by children in this study were relatively sparse, and do not reflect the spatial representation of children at these age (as demonstrated in other studies). This might be attributed to the instructions given to the participants, which focused solely on the route.

## 5. Conclusions

This study is the first to link between environmental walkability and spatial knowledge, a lacuna in the literature that was recently recognized [11]. Our findings suggest that children’s spatial knowledge is related to environmental walkability, but more significantly related to route distance, gender and school travel mode. According to the results, children’s spatial knowledge and representation of their h–s route was better in traditional compared to suburban neighborhoods, and these differences were attributed to several environmental attributes, including the walkability index, residential density, intersection density and land uses. Furthermore, our findings imply that walking to school may interact with environmental factors in shaping children’s spatial cognition. However, this assumption is yet to be tested, and future research with extended samples is needed.

### Implications for Urban Planning

The development of new neighborhoods in Israeli cities during the last two decades is guided by several common design principles, which were also employed in the planning of the suburban neighborhoods included in our study. These principles concern family-oriented environments, quality of life, cleanliness, safety and green open space (see Figure 1 and Figure 2). Implementation of these design principles is achieved through strict residential zoning and separation between car and pedestrian traffic (as illustrated in Figure 3d), which minimizes pedestrian traffic exposure and thereby allows children to walk freely in their home neighborhood environment (as illustrated in Figure 3d). Although such environments provide various advantages in terms of road safety, environmental quality and children’s outdoors opportunities, they create a rather dull and simplistic urban form. Indeed, the results of this research imply that the homogeneity and lower complexity of form in suburban neighborhoods yields lower capacity of spatial representation of that environment. This understanding is complementary to the notion that environments that are more differentiated, complex, and open to multiple manipulations (e.g., loose parts) are more intriguing to interact with and generally enhance residents’ sense of place and neighborhood satisfaction [57,58]. It seems that, in relation to the development of spatial knowledge, planning the new suburban neighborhoods has yet to better reconcile between residents’ clear preferences to green and vegetation in their environment [59,60] with their need for sense of safety and the need for a stimulating, interesting and activating environment that is currently better attained in the traditional neighborhood form and its new urbanism counterpart.

The growth in urban sprawl is particularly challenging in Israel due to its large and increasing population confined to the country’s limited land resources. Correspondingly, calls for sustainable development through urban sprawl mitigation has recently become more and more prominent in the Israeli planning discourse. This is clearly manifested in the national outline plan 35 that calls for urban densification through the re-development of existing settlements with an emphasis on compact urban design and mixed land uses [61]. Our findings support and extend these views by demonstrating how compact urban development may contribute especially to children. Following directly from our findings (Table 4), children’s wayfinding skills and active travel may be enhanced by planning strategies, such as: (1) Re-designing school registration zones to minimize distances between elementary schools and residential areas, (2) Ensuring that h–s shortest/direct routes pass through compact urban areas with high residential and intersection densities, and (3) ensuring that h–s shortest/direct routes pass through retail streets and/or include retail land uses en-route. Finally, in order to enhance the potential benefit of these planning principle, the findings reported in Section 3.3 suggest that environmental changes should be accompanied by community interventions to promote active travel to school. These recommendations, in addition to confronting sprawl-related problems, address current public health challenges in Israel, specifically—children’s physical inactivity, overweight and obesity [62,63].

## Figures and Tables

**Table 1:** List of dependent and independent variables and their sources.

	Conceptual Definition	Operational Definition	Data Source
**Dependent Variables**	Spatial declarative knowledge	Orientation and structure summary score	maps drawn by participants
		Richness summary score	maps drawn by participants
**Independent variables**	Neighborhood type	Traditional/Suburban	GIS
	Built environment en-route to school	Environmental variables along h–s route (within 25 m buffer): walkability index residential density intersection density % of land used for: retail, public institute, green open space.	GIS
	School travel mode	Walking to school most of the week (at least 3 days—yes/no)	Self-report
	Gender	Boy/Girl	Self-report

**Table 2:** Environmental variables as measured along the route to school (within a 25 m buffer).

	Overall Sample (N = 92)		Traditional Neighborhoods (N = 52)		Suburban Neighborhoods (N = 40)				
	Mean (SD)	IQR	Mean (SD)	IQR	Mean (SD)	IQR	T	Df	*p*
Walkability Index	0.28 (1.48)	−0.97–1.34	1.14 (1.18)	0.25–1.92	−0.83 (1.03)	−1.45–−0.35	**−8.00**	**90**	**<0.0001**
**Urban form measures**									
Street connectivity (intersections/sq km)	9.7 (5.13)	6.55–11.89	10.56 (3.93)	7.65–12.46	8.56 (6.24)	4.90–10.68	−1.67	90	0.18
Residential density (households/sq km)	15.44 (8.22)	7.11–21.76	21.61 (5.16)	18.19–23.94	7.42 (2.52)	5.51–9.79	**−16.45**	**90**	**<0.0001**
**Land use measures**									
% Retail area	0.05 (0.07)	0.00–0.10	0.08 (0.08)	0.01–0.12	0.00 (0.02)	0.00–0.001	**−5.11**	**90**	**<0.0001**
% Public Institute area	0.22 (0.15)	0.10–0.30	0.20 (0.11)	0.10–0.27	0.23 (0.19)	0.08–0.34	1.03	90	0.31
% Green Open Space	0.10 (0.12)	0.01–0.16	0.02 (0.04)	0.00–0.03	0.19 (0.13)	0.08–0.31	**9.25**	**90**	**<0.0001**

**Table 3:** Sketch map scores in the study sample by neighborhood type, school travel mode and gender.

	Total Sample (N = 92)	Neighborhood Type				School Travel Mode				Gender			
		Trad’ (N = 52)	Suburban (N = 40)	χ^2^	T	Walk (N = 52)	Other (N = 40)	χ^2^	T	Boy (N = 52)	Girl (N = 40)	χ^2^	T
**Orientation †**													
Inaccurately-oriented (0)	6 (7%)	3 (6%)	3 (8%)	1.99	NA	3 (5%)	3 (10%)	3.21	NA	1 (2%)	5 (10%)	2.45	NA
Partially-oriented (1)	28 (30%)	13 (25%)	15 (37%)			17 (27%)	11 (40%)			13 (29%)	15 (31%)		
Accurately-oriented (2)	58 (63%)	36 (69%)	22 (55%)			44 (68%)	14 (50%)			36 (67%)	29 (59%)		
**Structure**	1.36 (0–9)	0.83 (0–5)	2.05 (0–9)	NA	−3.41 *	0.95 (0–5)	2.29 (0–9)	NA	3.46 *	0.95 (0–5)	1.71 (0–9)	NA	2.06 *
Gap in segments [M(range)]													
**OR**													
Perfect match (no gap)	38 (41%)	32 (62%)	6 (15%)	20.20 ***	NA	32 (50%)	6 (21%)	6.56 *	NA	19 (44%)	19 (39%)	0.28	NA
Gap of 1 segment or more	54 (59%)	20 (38%)	34 (85%)			32 (50%)	22 (79%)			24 (56%)	30 (61%)		
**Summary score**	8.23 (1–10)	8.81 (3–10)	7.48 (1–10)	NA	3.46 **	8.69 (3–10)	7.18 (1–10)	NA	3.66 **	8.70 (3–10)	7.82 (1–10)	NA	−2.22 **
orientation and structure [M(range)]													
**Richness**													
**Diversity**	2.23 (0–6)	2.15 (0–6)	2.33 (0–5)	NA	−0.64	2.11 (0–5)	2.50 (1–6)	NA	1.44	1.98 (0–4)	2.45 (0–6)	NA	1.81 *
Number of themes [M(range)]													
**Level of detail**	3.35 (0–11)	3.29 (0–11)	3.43 (0–7)	NA	−0.30	3.29 (0–11)	3.43 (1–10)	NA	0.3	2.91 (0–7)	3.73 (0–11)	NA	1.89 *
Number of elements [M(range)]													
**Summary score**	5.58 (0–16)	5.44 (0–16)	5.75 (0–11)	NA	−0.45	5.23 (0–13)	6.36 (2–16)	NA	1.57	4.88 (0–11)	6.18 (0–16)	NA	1.96 *
Diversity and detail [M(range)]													

* *p* < 0.01, ** *p* < 0.001, † 0 = inaccurate orientated map (in terms of both top/down and right/left literalities), 1 = partially oriented map (one laterality is accurate (either top/down or right/left)), 2 = accurate orientated map (in terms of both top/down and right/lest literalities).

**Table 4:** Correlations between sketch maps’ summary scores and objective environmental measures en-route (Pearson coefficient, N = 92).

		Sketch Maps’ Summary Scores-			
		Orientation and Structure		Richness	
		R	*p*	R	*p*
**Walkability index**		0.40 ***	**0.000**	−0.03	0.81
**Urban form**	Residential density	0.35 *	**014.0**	−0.12	0.25
	Intersection density	0.30 ***	**000.0**	−0.02	0.86
**Land use**	% retail	0.22 *	**03.0**	0.08	44
	% public institutes	−0.009	0.09	0.07	52
	% green open space	−0.21 *	**0.03**	0.05	62

* *p* < 0.05, *** *p* < 0.0001.

**Table 5:** Multivariable linear regression models to predict the orientation and structure summary score.

Independent variables	Orientation and Structure Summary Score	
	β	*p*
Route distance	**−0.33**	**0.005**
Gender (boy vs. girl)	**0.23**	**0.022**
Walking to school (number of days per week)	**0.20**	**0.05**
Neighborhood type	0.06	0.60
Model summary	R² = 0.30, *p* < 0.0001, N = 92	
